# Extending decision making competence to special populations: a pilot study of persons on the autism spectrum

**DOI:** 10.3389/fpsyg.2015.00539

**Published:** 2015-04-28

**Authors:** Irwin P. Levin, Gary J. Gaeth, Megan Foley-Nicpon, Vitaliya Yegorova, Charles Cederberg, Haoyang Yan

**Affiliations:** ^1^Department of Psychology, University of Iowa, Iowa City, IA, USA; ^2^Department of Marketing, University of Iowa, Iowa City, IA, USA; ^3^Department of Psychological and Quantitative Foundations, University of Iowa, Iowa City, IA, USA; ^4^Department of Social Work, University of Iowa, Iowa City, IA, USA; ^5^Department of Psychology, University of Michigan, Ann Arbor, MI, USA

**Keywords:** decision making, persons on the autism spectrum, risk taking, perception of social norms, framing effects

## Abstract

The area of decision making has much to offer in our effort to understand special populations. This pilot study is an example of just such a project, where we illustrate how traditional decision making tools and tasks can be used to uncover strengths and weaknesses within a growing population of young adults with autism. In this pilot project we extended accounts of autistic behavior such as those derived from “theory of mind” to predict key components of decision making in high-functioning young adults on the autism spectrum. A battery of tests was administered to 15 high-functioning college students with autism spectrum disorder (ASD), focusing on decision making competence (DMC) and other aspects of decision making related to known deficits associated with autism. Data from this group were compared to data from unselected college students receiving the same measures. First, as a test of a key social deficit associated with autism, the target group scored much lower on the Empathy Quotient scale. Traditional elements of decision making competency such as Numeracy and application of decision rules were comparable across groups. However, there were differences in thinking style, with the ASD group showing lesser ability and engagement in intuitive thinking, and they showed lower levels of risk taking. For comparisons within the ASD group, autobiographical reports concerning individual lifestyles and outcomes were used to derive a scale of Social Functioning. The lowest scoring individuals showed the lowest levels of intuitive thinking, the lowest perceived levels of others’ endorsement of socially undesirable behaviors, and the lowest ability to discriminate between “good” and “bad” risks. Results are discussed in terms of interventions that might aid high-functioning young adults with ASD in their everyday decision making.

## Introduction

The primary goal of this pilot study is to demonstrate how decision making research in general, and research on decision making competence (DMC) in particular, can advance our understanding of young adults who are high functioning on the autism spectrum. This group is important to study from a practical perspective because of the increasing number of people diagnosed with autism spectrum disorder (ASD), and because these young adults are at a time in their development when life-altering decisions need to be made. We start by focusing on the key concepts of theories of autism and generating novel predictions of how they apply to decision-making behavior. We then review literature examining ASD from a clinical, applied perspective to generate a series of research questions and hypotheses, and provide preliminary tests in a pilot study.

We feel that recent research on DMC shows great promise as a tool to better understand the strengths and weaknesses of special populations. First, it represents a subtle shift from earlier work on biases and heuristics in emphasizing the positive rather than the negative side of decision making. Second, DMC research often focuses more on the individual decision maker and his or her characteristics, and uses within-subject measures. Third, this research has emphasized the need to assess the validity of standard laboratory tasks in predicting real-world decision making and its consequences (Parker and Fischhoff, 2005; Del Missier et al., 2012; Weller et al., 2012). We feel that these developments show great promise for uncovering both the strengths and weaknesses of special groups of decision makers such as those with ASD. We start with a brief summary of clinical work describing the characteristics of the disorder and follow this with our proposed linkages to decision making processes.

Autism spectrum disorder is a term representing a multifaceted and diverse set of related neurodevelopmental conditions where individuals exhibit social communication and behavioral difficulties (Tager-Flusberg, 2007; American Psychiatric Association [APA], 2013). Persons with ASD have extremely variable cognitive and behavioral functioning abilities and this influences how the core diagnostic symptoms manifest. There currently is no one theory available to explain the full range of phenotypic presentations (Tager-Flusberg, 2007; Van de Cruys et al., 2014). However, one theory in particular, “theory of mind” (Baron-Cohen et al., 1985), postulates that individuals with ASD display an impaired capacity to understand the feelings, thoughts, intentions, beliefs, and potential behavioral reactions of others. Nevertheless, not all individuals with ASD fail classic theory of mind tasks, such as the false-belief task (i.e., a test of one’s ability to take the perspective of someone else), leading some to suggest that basic perspective-taking may develop later in those with ASD, More complex aspects, such as understanding accidental harm, may never fully develop, even in higher functioning populations (Van de Cruys et al., 2014). Similarly, those who are older or have higher cognitive functioning may pass the theory of mind tests by relying on language and other non-social cognitive functions rather than on social insight to solve the problems (Frith and Frith, 2003).

It has been proposed that the theory of mind construct can be divided into cognitive and affective abilities that are distinguished by two distinct neural networks (Shamay-Tsoory et al., 2003; Shamay-Tsoory and Aharon-Peretz, 2007). Cognitive theory of mind deficits include challenges associated with cognitive flexibility and perspective taking. Affective theory of mind deficits include the capacity to understand and recognize the emotional experiences of others, or the ability to experience empathy. In high-functioning individuals with ASD, the decreased ability to process the emotional components of social information is independent from the person’s normal or above normal cognitive functioning (Buitelaar and van der Wees, 1997). Given this information, scholars suggest that tasks be expanded to include a wider and more realistic range of social situations to demonstrate how theory of mind manifests differently as one develops (Baron-Cohen et al., 1997; Tager-Flusberg, 2007; Scheeren et al., 2013). In the current study we provide measures of thinking style, perception of social norms, risk-taking, and reacting to affective cues.

To elaborate on how these concepts relate to DMC, assessing thinking style of adults with ASD is consistent with the notion that understanding another’s perspective involves general deductive reasoning (McDonald and Flanagan, 2004) and executive functioning skills, which include decision-making (e.g., Carlson et al., 1998; Carlson and Moses, 2001; Perner et al., 2002; Talwar and Lee, 2008). Furthermore, some limited data suggest that adults with ASD may underestimate others’ perceptions of what kinds of negative behaviors are acceptable. For example, in Moran et al. (2011) study of moral judgment in adults with ASD and neurotypical adults, the ASD group failed to differentiate accidental from intentional harms toward others. The authors suggested that the participants with ASD over-relied on outcomes of actions and under-relied on intention.

Also central to DMC and its relationship to theory of mind deficits in ASD is the commonly observed strict adherence to rules and inflexibility in the presence of new and conflicting information (American Psychiatric Association [APA], 2013). A related finding is that persons with ASD tend to take longer to make decisions and are more likely to report avoidance of decision-making (Luke et al., 2012; Brosnan et al., 2014). When individuals are faced with risky situations, they may struggle to take a risk if it involves their needing to make a rule violation. That is, individuals need to both learn from their errors and know when to ignore errors (Van de Cruys et al., 2014). The inability to be flexible may translate into difficulty taking risks, even when doing so would be beneficial. Finally, previous researchers have found that emotional recognition and theory of mind are correlated such that individuals with ASD have significantly lower cognitive and affective empathy than have those without ASD (Mathersul et al., 2015), and this may impact their ability to make decisions that involve the interpretation of subjective information.

In addition to establishing a theoretical rationale for selecting our target group, a major reason why we focused on young adults with ASD is that the unemployment rate in this group is disturbingly high (Ballaban-Gil et al., 1996; Howlin et al., 2004; Taylor and Seltzer, 2011; Roux et al., 2013; Burgess and Climera, 2014), even in light of advanced degree obtainment (Baldwin et al., 2014). On the other hand, there are individual examples of great success such as the extraordinary contributions in animal science by Temple Grandin (Kalbfleisch, 2013). Prior research and anecdotal reports have suggested that individuals with ASD have several unusually strong skills, such as task endurance and high intensity focus. It is clear that advances in understanding individuals with high-functioning ASD in particular require a balanced view of ways to build on strengths and overcome weaknesses. The measures of DMC are well-positioned to do this.

Recent work by members of our research team and others has extended the study of DMC in several ways: (1) sampling across the age spectrum to demonstrate that the development of DMC is “an act in progress” (Weller et al., 2012); (2) using a longitudinal research design to establish the stability of DMC as an individual differences measure that captures real-world decision making and its consequences (Weller et al., 2015); and (3) identifying within and across age groups those individual dispositional characteristics that account for variance associated with different components of DMC (Levin et al., 2014). Levin et al. (2014) showed that specific individual difference variables were related to different components of DMC, for example, surgency and the ability to discriminate between good and bad risks.

There has been some limited research on how adults with ASD differ from controls on specific decision making tasks. For example, in a study by De Martino et al. (2008) employing a gambling task, ASD subjects showed a reduced susceptibility to framing effects as measured by smaller differences than control subjects in risk-taking to achieve gains and risk-taking to avoid losses. Furthermore, in support of the proposition that ASDs fail to incorporate emotional context into the decision process, De Martino et al. (2008) provided evidence tying insensitivity to contextual frame to psycho-physiological evidence (skin conductance response).

Interestingly, this diminished reaction represents an advantage to persons with ASD because *resistance to framing effects* has been considered as a positive component of DMC. However, in other contexts, diminished emotional processing can be a deterrent to constructive decision making, especially when learning from experience. Morsanyi and Holyoak (2010) compared children with ASD and typically developing children on a battery of analogical reasoning tasks and found no group differences, suggesting that the ability to reason systematically with relations is intact in children with ASD. In the present study we separate measures of analytic/logical thinking and intuitive/experiential thinking. If intact reasoning persists until early adulthood, we would expect no difference between our ASD and control groups on our measure of analytic/logical thinking.

In an experiential decision making task, the Iowa gambling task (IGT), Johnson et al. (2006; see also Yechiam et al., 2005) showed that individuals with ASD were less responsive to choice outcomes than were controls, and were more influenced by the attraction of previously unexplored and irrelevant options. Our measure of thinking style that separates intuitive/experiential thinking from analytic/logical thinking is consequently expected to show lower levels of the former in the ASD group.

Also of interest is decision making under risk because so many of the life-altering decisions facing high-functioning young adults with ASD involve the element of risk. For example, “If I take a full course load, I will graduate sooner but will the pressure be too great for me?” “Should I move away from my comfort zone at home to where new opportunities will open up for me, but where I may not be able to function in a new social environment?” “Should I take a lower paying job that will allow me to avoid extensive interpersonal contact or should I reach for a higher paying job that requires more interpersonal encounters?”

A recent study by South et al. (2011) compared children and adolescents with ASD to age-matched controls on an experimental risk-taking task, the Balloon Analog Risk Task. They found no difference between groups in the summary measure of risk-taking. However, they found interesting differences in the mediators of risk-taking, leading them to conclude that task performance was motivated by fear of failure in the ASD group and by sensitivity to reward in the control group. One shortcoming that they pointed out was the inability to examine responses to gain and loss trials separately. Our use of multiple tasks will include separation of risk-taking to achieve gains and risk-taking to avoid losses. However, their use of individual difference measures to examine within-group as well as between-group differences is consistent with the current approach to help inform individualized interventions for persons on the spectrum.

### Research Questions and Predictions

Based on the theoretical accounts and prior studies described above, we formulate the following set of research questions. In some cases prior thinking leads us to specific predictions for our measures; in other cases the questions are open-ended.

1. How do the ASD and control groups compare on Recognizing Social Norms, a set of measures designed to assess the extent to which individuals personally endorse socially undesirable behaviors and the extent to which they perceive others to endorse such behaviors? Based on theory of mind conclusions concerning ASD deficits in understanding others’ feelings and intentions, we expect differences in these measures but leave the direction of difference as an empirical question.
2. How will the ASD group and controls compare on framing effects? Based on models of autistic behavior that focus on lowered responsiveness to emotional cues and the findings of De Martino et al. (2008), we predict lower framing effects in the ASD group.
3. How do the groups compare on Thinking Style, a set of measures designed to distinguish analytic/logical thinking style from intuitive/experiential thinking? Based on earlier results showing lowered levels of learning from experience and increased decision times (Luke et al., 2012; Brosnan et al., 2014), we predict that our ASD group will be lower on measures of intuitive/experiential thinking but not on measures of analytic/logical thinking.
4. How do the groups compare on riskiness of decisions, separating risks to achieve gains and risks to avoid losses? South et al. (2011) concluded that in dealing with risk, those with ASD compared to controls were more motivated by fear of failure and less by sensitivity to reward. We provide a variety of measures to examine this question.
5. How do individuals with high-functioning ASD compare with controls on measures such as Numeracy and Use of Decision Rules, which are thought to represent cognitive ability and use of cognitive resources? We have no reason to expect group differences on these measures but their inclusion may help to predict performance on more complex measures of decision making.

In addition to addressing these five questions by considering ASD-control group differences, we provide individual difference analyses to examine the extent to which crucial social functioning within the ASD group varies as a function of the same factors that differentiate ASDs from controls.

## Methods and Procedure

### Participants

As part of what we consider a pilot study, we compared a group of 15 college-aged students with ASD (13M, 2F; age *M* = 23.92, SD = 4.36, range = 18–30) with unselected students for whom we had prior data on several measures that are used in DMC measurement. In addition, we assessed through a series of scales and interviews, the personal and interpersonal aspects of the lives of our participants with ASD. The combination of these objective and subjective measures obtained with restricted sampling from a relatively rare population required face-to-face data collection spaced across two sessions. Thus, in contrast to typical judgment and decision making research, our emphasis was on collecting in-depth individual data at the expense of a large sample size.

The following is the series of checks and balances used in recruitment and ASD diagnostic verification:

1. Initial recruitment was accomplished through a notice, approved for standards of ethics by the university’s institutional review board (IRB), that was sent to all members of a large Midwestern university describing the need for young adults, ages 18–28, who have been diagnosed with ASD to participate in a study of decision making. (IRB approval was given for all the control subject procedures as well.) Those interested in participating were told to express their interest through a response to this email that included their telephone number. Following the general mailing, targeted mailings were sent to offices on campus that worked with individuals with ASD.
2. A follow-up call was made to interested persons reiterating that they would be participating in a study of decision making for adults on the autism spectrum. A few sample questions were asked. If the answers reflected understanding of the questions and if the person verified his or her diagnosis, the participant was recruited for two sessions in the laboratory. Each lasted from 1 h to one and a half hours for which compensation was provided (a $25 gift card for completing each session but they were not told this before coming).
3. When participants arrived in the lab for the first of two sessions, they read through and signed the informed consent that again described that they were participating in the study because they “have been identified as on the autism spectrum.”
4. In order to qualify for inclusion in the ASD group, at the beginning of Session 1 potential participants were required to complete an oral interview to affirm diagnosis. Specifically, they were asked to provide details of how and when they were diagnosed. We employed this strategy given that those with ASD tend not to lie (e.g., Baron-Cohen, 2008; Jaarsma et al., 2012). Here are some sample responses: “Mild Aspergers, was misdiagnosed in middle school w/ full Aspergers, was on high dose of Strattera, was re-diagnosed after high school. Understood it better.” “Younger autistic tendencies, later it was Aspergers. First time was 4 years old, reevaluated when came to Iowa. In 5^th^ grade it was Aspergers.” “Aspergers syndrome. Officially in 2004. However, parents suspicious of ASD much earlier than that. Parents suggested an evaluation. Saw a psychiatrist.”

### Tasks and Measures

Fundamental to the goal of understanding the strengths and weaknesses of research participants is collecting information on their DMC. For present purposes of addressing our research questions about persons with ASD, we included the following basic measures of DMC: Numeracy, Applying Decision Rules, Recognizing Social Norms, and framing effects. We also included the Cups Task of risky decision making that assesses the ability to discriminate between “good risks” in which the expected value (EV) favors a risky choice over a riskless choice and “bad risks” where the EV favors the riskless choice over the risky choice (Levin et al., 2007). We describe each measure in more detail below (see also Weller et al., 2012, Appendix 1). Added to these decision making assessments, we included measures of the rational-experiential inventory (REI, Epstein et al., 1996) of thinking style, and the Empathy Quotient scale (EQ; Baron-Cohen and Wheelwright, 2004) often used to assess deficits in the processing of social information among persons with ASD.

As an original contribution arising from our use of face-to-face interview techniques, we use open-ended questions focusing on participants’ social experiences concerning living relationships: the quality of personal relations at home, school and work; and friendship networks. Participants were asked how satisfied they were in each of these domains and were encouraged to elaborate on any difficulties they were experiencing. Transcripts were formed and scored by two independent coders on the quality of social relations, using a 1–5 scale where higher numbers represented better relations. The two raters were never more than one point apart and the sum of their scores was used as an individual difference measure of social and interpersonal success abbreviated here as “social functioning.”

#### Numeracy

Numeracy is a measure of the understanding and competence a decision maker has in using numerical information (Lipkus et al., 2001). Some sample items are: “If the chance of getting a disease is 10%, how many would be expected to get the disease out of 1000?” “A bat and a ball cost $1.10 total. The bat costs a dollar more than the ball. How much does the ball cost?” “In a lake there is a patch of lily pads. Every day the patch doubles in size. If it takes 48 days for the patch to cover the entire lake, how long would it take for the patch to cover half the lake?” There are 8 items in total with number correct as the coded score. This scale overlaps with another popular scale of cognitive ability, the cognitive reflection test (CRT; Frederick, 2005).

#### Applying Decision Rules

This is a measure of the ability and effort employed to utilize information of varying complexity in a decision matrix to make the best choice in each of a series of choices between video game systems. There are seven items in total, ranging from simple to complex, scored for number correct (Payne et al., 1993; Bruine de Bruin et al., 2007). For each question, participants were asked to think about how each person makes his or her choice, and then pick the DVD player they choose. Each of the five options was given a value of 1 (very low), 2 (low), 3 (medium), 4 (high), or 5 (very high) on each of four attributes displayed in matrix form. An example of a relatively simple question is, “Which one of the DVDs would Brian prefer if Brian selects the DVD player with the highest number of ratings above Medium?” Only one option clearly met this criterion and it only required counting the number of values above 3 in each row. An example of a more difficult question is, “Tyrone wants a DVD player that either has a Very High rating for Programming Options, or one that scores at least Medium on every feature. Which three of the presented DVD players would Tyrone prefer?” Answering this question requires attention to more than one criterion.

#### Framing Susceptibility Measures

Based on a typology of framing effects developed in our prior work (Levin et al., 1998, 2002, 2014), different types of framing conditions are generated by different underlying mechanisms. For the purposes of this research, two types of framing are included and analyzed separately. *Attribute framing* involves describing an attribute of an object or event in objectively equivalent, but affectively distinct, terms (e.g., 85% lean ground beef versus 15% fat ground beef). The impact of the attribute framing effect is measured as the mean difference in ratings, such as quality, between the positive and negative conditions on a 1–6 scale averaged over three items. *Risky choice framing* is determined by presenting two choice options that differ in risk-level and that are alternatively framed in terms of gains or losses (Tversky and Kahneman’s 1981 Asian disease problem using lives saved or lives lost). The framing effect is measured by the difference in ratings between the loss and gain versions of the problem [on a 1–6 scale ranging from “much prefer A” (the riskless option) to “much prefer B” (the risky option)]. For both attribute framing and risky choice framing, one version of a problem is presented in session 1 and the second version is presented in session 2, with the different versions randomly assigned to sessions. Smaller differences represent lesser Framing Effects.

#### Cups Task: Sensitivity to Risk

To measure the decision makers’ sensitivity to risk, we used the “Cups Task” (Levin et al., 2007). The Cups Task was developed to separate risk-taking for gains and losses of monetary currency and to measure the ability to discriminate between “good risks” where the EV is more favorable for the risky option than the riskless option and “bad risks” where the EV is less favorable for the risky option than the riskless option. On each trial, the participant chooses between an array of cups with a fixed outcome and an array of cups with uncertain outcome. An example of a “gain” trial would be a choice between a sure gain of one quarter versus a chance of winning 2, 3, or 5 quarters under one (of three) cups or winning 0 quarters under the remaining two cups. A corresponding example of a “loss” trial would be the choice between a sure loss of one quarter and a chance of losing 2, 3, or 5 quarters under one (of three) cups or losing 0 quarters under the remaining two cups. For each type of trial a 3 × 3 design was used where the number of cups was 2, 3, or 5 and the number of quarters to be won (or lost) by a risky choice was 2, 3, or 5. Some of these combinations lead to a greater EV for the risky choice and some lead to greater EV for the riskless side. There were three trials of each combination. A primary dependent measure is the difference between the number of risky choices made for “good” or what we call “risk advantageous” (RA) trials and “bad” or what we call “risk disadvantageous” (RD) trials. Higher differences (RA-RD) represent greater discrimination of good and bad risks.

#### Recognizing Social Norms

The recognition or perception of social norms is an assessment based on responses across the two sessions (Jacobs et al., 1995). In one session the participant indicated the likelihood of personally saying that it is okay to perform a set of 15 socially undesirable acts, ranging from not being on time for an appointment, not returning a borrowed item, and keeping things found in the street, to drunk driving, using violence to solve an argument, and not telling the police after witnessing a crime. In another session, the participant estimated the percentage of his or her peers who would say it is okay to perform the same act. We felt that this measure might partially capture a deficit in social perception in terms of what is socially acceptable and what is not.

#### Rational-Experiential Inventory

This dual component scale is used to measure preferences in the way we process information with a focus on the degree to which a person uses analytic and logical thinking or the extent to which he or she uses intuition and personal experience to make decisions (Epstein et al., 1996; Pacini and Epstein, 1999). Rationality is measured by the Need for Cognition scale (e.g., “I prefer complex over simple problems.”) and Experientiality by the Faith in Intuition scale (e.g., “I trust my initial feelings about people.”). The rationality component is measured with two subscales, Rational Ability, which reflects the ability to think logically and analytically and Rational Engagement, which reflects reliance on and enjoyment of thinking in an analytical, logical, manner. The Experientiality component is also measured with two subscales, Experiential Ability, which is the ability to use intuitive impressions and feelings, and Experiential Engagement, which is the reliance on and enjoyment of feelings and intuitions in making decisions. Pacini and Epstein (1999) demonstrated the discriminant validity of their two components of REI by showing that the two subscales were independent of each other, each was correlated with different components of the Big Five personality scales, and that the two scales were differentially related to responding in a game of chance.

#### Empathy Quotient

This scale measures the degree to which a person shows empathy (Baron-Cohen and Wheelwright, 2004). The scale contains 60 items, 40 of which are relevant and 20 of which are distractors. Each of the relevant items was scored as 1 or 2 points depending on whether the response was “definitely agree/disagree” or “slightly agree/disagree.” This scale is often used to screen individuals for ASD and was originally developed for the Autism Research Centre by Simon Baron-Cohen and Sally Wheelwright (Wheelwright et al., 2006). Examples include, “I try to solve my own problems rather than discussing them with others,” “I am good at predicting how someone will feel,” and “Friendships and relationships are just too difficult.” Scores can range from 0 to 80 (most empathetic). A study by Dawda and Hart (2000) examined reliability and validity of the Bar-On Emotional Quotient Inventory (Bar-On, 1997, 2004) in a sample of 243 university students. Results indicated that the EQ domain and component scales had good item homogeneity and internal consistency. Scores were not unduly affected by response styles or biases. They reported a meaningful pattern of convergent validities with respect to measures of normal personality, depression, somatic symptomatology, intensity of affective experience, and alexithymia.

## Results

We divide our analyses into two parts: comparisons between participants with ASD (ASD group) and college-age controls, and comparisons within the ASD group. The former will allow us to compile profiles of strengths and weaknesses of young adults with ASD in comparison to controls. The latter will allow us to examine individual differences within the ASD group that impact decision making performance.

### Between-group Comparisons

Because our goal is to compare the decision making profiles of young adults with ASD and controls, and because our measures did not match one-for-one with those used in prior DMC research, we provide separate rather than combined measures. As a preliminary replication of a well-established result, we compare EQ scores between groups where low EQ is a signature index of ASD. Scores were in fact quite disparate between groups with little overlap [*M*_ASD_ = 27.87, SD = 8.85; *M*_Control_ = 43.65, SD = 9.60; *M*_diff_ = –15.78, *t*(68) = –5.73, difference *p* < 0.0001].

In the first formal set of analyses, we concentrate on those measures that represent “cognitive” processes plus thinking style. These comparisons are summarized in Table 1 where different sample sizes for the control group arise from different available data sets of college students for the different measures. These include the following, which have traditionally been considered as components of DMC: Numeracy, Applying Decision Rules, and Framing Effects. Based on recent research in our laboratory, attribute framing effects and risky choice framing effects represent different balances of cognitive and affective processes, so we consider them separately (Levin et al., 2014). Also included are the REI measures of thinking style and measures of performance on the Cups Task, which has the unique features of separating risk-taking to achieve gains and risk-taking to avoid losses, as well as the ability to discriminate “good” and “bad” risks within the gain and loss domains.

**Table 1:** **Means and *t*-tests comparing ASD participants with “controls” on cognitive measures**.

**Measure (upper/lower limits)**	**ASD (*N* = 15) mean (SD)**	**Control mean (SD) (Sample)**	*t*-value (*p*)*
Numeracy (0–8)	5.20 (1.97)	4.88 (1.64) (*N* = 211)	0.72 (0.472)
Applying decision rules (0–7)	5.06 (1.49)	4.41 (1.68) (*N* = 211)	1.46 (0.146)
Attribute framing effect (–5 to 5)	0.46 (0.46)	0.33 (0.75) (*N* = 131)	0.96(0.348)
Risky choice framing effect (–5 to 5)	0.33 (1.29)	0.80 (2.10) (*N* = 131)	–1.24 (0.229)
Rational ability (0–10)	3.70 (0.80)	3.79 (0.54) (*N* = 80)	–0.42 (0.681)
Rational engagement (0–10)	3.78 (0.83)	3.51 (0.62) (*N* = 80)	1.46 (0.147)
Experiential ability (0–10)	2.80 (0.85)	3.43 (0.51) (*N* = 80)	–2.78 (0.014)
Experiential engagement (0–10)	2.94 (0.62)	3.43 (0.52) (*N* = 80)	–3.25 (0.002)
Cups task: risk taking on gain trials	12.60 (5.38)	16.95 (6.77) (*N* = 358)	–2.46 (0.01)
Cups task: risk taking on loss trials	14.33 (7.02)	18.08 (5.93) (*N* = 358)	*–*2.38 (0.02)
Cups task: RA-RD on gain trials	1.33 (3.46)	2.59 (2.89) (*N* = 358)	*–*1.64 (0.10)
Cups task: RA-RD on loss trials	2.00 (2.53)	1.67 (2.91) (*N* = 358)	0.43 (0.666)

*t-tests were adjusted for unequal variance where appropriate.

Individuals in the ASD group were comparable to controls on the traditional measures of DMC, and even tended to be a bit higher on the use of decision rules in decision matrices. The two groups were also comparable on Framing Effects. While the two groups did not differ significantly in the magnitude of Framing Effects, these effects served as important individual difference variables within the ASD group. This will be discussed in more detail later.

Robust differences in decision style were revealed by the REI. Participants in the ASD group showed less ability and engagement than controls in experiential/intuitive thinking style. No differences were found in rational/deliberative thinking. This set of results suggests that our target group was less likely to make decisions based on emotion and intuition but not less likely to make decisions based on well thought-out deliberations and calculations.

Performance on the Cups Task likewise revealed some important group differences. The ASD group made significantly fewer risky choices on both trials to achieve gains and on trials to avoid losses. However, the participants in the ASD group were equally as likely as controls to discriminate risk-advantageous and risk-disadvantageous choices on loss trials but were marginally poorer on gain trials.

Table 2 focuses on behavioral and social measures: components of Recognizing Social Norms where for each of a series of socially-undesirable acts, participants rate their personal likelihood of saying it is okay to perform each act and estimate the percentage of their peers who would say it is okay. Overall, participants with ASD, compared to controls, gave lower personal endorsements of behaviors that violate social norms. They also tended to give lower ratings of others’ endorsements of these behaviors but this effect failed to reach statistical significance. At the individual item level, participants in the ASD group gave significantly lower ratings than controls for keeping things that don’t belong to you, not holding the door open for someone, not being punctual, and not returning calls. Given that participants in the ASD group were more consistent in self-other judgments than were controls, their perceptions of others’ undesirable behaviors warrants further study.

**Table 2:** ***t*-test results comparing ASD participants with “controls” on social measures**.

	**ASD (*N* = 15)**	**Control **	***t*-value (*p*)**
Self-endorsement of Social norms (0–1)	0.38 (0.26)	0.61 (0.20) (*N* = 174)	4.17 (<0.0001)
Perception of others’ endorsement of Social norms (0–100)	35.69 (17.12)	42.20 (14.11) (*N* = 174)	1.69 (0.0938)
Difference between self-endorsement and perceived others’ endorsement of Social norms (0–1)	0.02 (0.22)	0.19 (0.30) (*N* = 174)	2.14 (0.03)

### Within-group Comparisons

Here the focus was to determine the extent to which differences within the ASD group on performance measures can be captured by dispositional measures. In spite of the common label of ASD, the relatively small sample size and the restricted range of scores on scales such as EQ, some interesting inter-correlations were observed between the various measures. Table 3 focuses on relations between behavioral measures and their predictors. We judge the most important results to be those related to our original “social functioning” measure of how well each person with ASD functions in the social world based on self-reports of relations at school, at work, at home, and in presence/absence of friends. Social functioning was lowest for those showing the lowest levels of engagement in experiential/intuitive decision making style, those showing the lowest levels of discrimination between “good” and “bad” risks but only in the gain domain of the Cups Task, and those showing the largest risky choice Framing Effects. Personal endorsement of negative social behaviors and perceptions of endorsement by others were highest for those showing the highest levels of rational ability and rational engagement, and lowest for those exhibiting the highest risky choice Framing Effects.

**Table 3:** **Pearson correlations within ASD participants**.+

**|**	**Social functioning**	**Social norms-self**	**Social norms-other**
Numeracy	–0.055	0.432	0.425
Decision Matrix	–0.306	0.440	0.070
EQ	0.256	0.027	–0.420
Rational ability	0.298	0.526*	0.533*
Rational engagement	0.458	0.614*	0.552*
Experiential ability	0.272	0.100	0.004
Experiential engagement	0.575*	–0.030	–0.036
Attribute framing effect	0.501	–0.278	0.200
Risky choice framing effect	–0.747**	–0.541*	–0.538*
Risk in gain	0.06203	–0.263	–0.413
Risk in loss	0.17638	–0.185	–0.196
RA-RD in gain	0.676**	0.508	0.433
RA-RD in loss	0.081	0.365	0.336

^+^All significant correlations were reviewed to ensure a linear relationship. *p < 0.05, **p < 0.01.

Although not shown in Table 3, those scoring lowest on social functioning were also lowest on perceptions of others’ endorsement of negative social behaviors (*r* = 0.56, *p* = 0.03) but not significantly lower on personal endorsement of negative social behaviors (*r* = 0.31, *p* = 0.27).

## Discussion

### Summary of Key Results

We first summarize what we consider to be the most revealing results emerging from our pilot study comparing high-functioning ASD and young adult controls, organized around our original research questions. These in turn were largely theory-driven, motivated by the constructs of theory of mind.

RQ1: Consistent with theory of mind notions that persons on the autism spectrum have difficulties perceiving social cues, individuals in the ASD group were less apt than controls to endorse undesirable social behaviors and they tended to be less likely to perceive others as endorsing undesirable social behaviors. The latter was especially true of those with the poorest social functioning. Interestingly, individuals in the ASD group showed a significantly greater degree of coherence between personal endorsements and perceptions of others.
RQ2: Here we predicted lower Framing Effects for the ASD group. This was not confirmed but Framing Effects emerged in within-group analyses (see below).
RQ3: As predicted, participants in the ASD group had lower levels of ability and engagement in experiential/intuitive thinking style but showed no differences in analytic/logical thinking.
RQ4: Participants with ASD were less risk-taking than controls but were not significantly different from controls in the ability to discriminate between good and bad risks. There was, however, a marginal tendency for the ASD group to be less discriminating on gain trials.
RQ5: The ASD group scored at least as well as controls on the cognitive measures of DMC, namely Numeracy, and Applying Decision Rules.

In addition to these tests of differences between groups, within the ASD group, poor social functioning was related to less engagement in intuitive thinking styles, poorer ability to discriminate between good and bad risks, but only for risks to achieve gains, and greater risky choice Framing Effects.

### Clinical Implications and Applications

Few researchers have examined decision-making in young adults in general (Luke et al., 2012), and fewer seek to improve decision-making skills in the ASD population. While initial interventions are being developed to help young adults with ASD gain employment-seeking skills (Strickland et al., 2013), there is a dearth of research examining how improvements in basic decision-making abilities and/or inabilities may impact an ASD individual’s employment or achievement success. However, these are critical skills for young adults and students with ASD who need to make good decisions to be successful in life. Additionally, social skills curriculums exist for young adults with ASD (e.g., Baker, 2003; Laugeson and Frankel, 2010) but none are empirically based or take decision-making strengths and impairments into consideration.

Here we discuss potential clinical applications of our findings contingent on the ability to overcome the limitations discussed below and replicate these findings with larger sample sizes and a greater array of diagnostic tools. For example, risk taking is an aspect of decision-making that is complex and highly dependent on both the situation and individual; thus it is clinically relevant. It may be that the nuances involved in determining what situations call for what decisions is lost on the person with ASD; thus, the individual may be less likely to take risks that would be to their advantage. Present results show that the poorest functioning ASDs are less apt to discriminate between “good” and “bad” risks involving possible gains. A potentially useful intervention here would be to give them examples where taking a risk that may at first seem undesirable (going for a job interview that is uncomfortable and with uncertain outcome) can lead to long-term gains (financial security).

Second, our results suggest that decisions that normally rely on affect or intuition may be more difficult for individuals with ASD to make than decisions that rely on deliberative and logical thinking (see also Yechiam et al., 2005; De Martino et al., 2008; South et al., 2008, 2011). A potentially useful intervention here would be to provide examples where excessive deliberation can be counter-productive such as selecting a place to go out for dinner or something to wear to work.

Previous research supports the notion that individuals with ASD are good rule-followers (Attwood, 1998; Hill, 2004; Sterponi, 2004; Shulman et al., 2012), which is consistent with the current results indicating that individuals with ASD performed well on the Applying Decision Rules and Numeracy measures and most were able to discriminate between good and bad risks. The present findings suggest they typically do not endorse undesirable social behaviors, which is positive, but they may be unrealistic in recognizing these behaviors in others, which could be problematic. These identified challenges are consistent with global deficits many individuals with ASD encounter related to theory of mind (Baron-Cohen et al., 1994, 1997). Individuals with ASD are at an increased risk for being taken advantage of in social and professional settings (Hillier et al., 2007; Wilczynski et al., 2013), which can be an additional focus of therapeutic intervention. Use of role-play, rehearsal, and problem-solving can help a therapist capitalize on the client’s personal ethical code and increase his or her recognition of when their personal standards are or are not present in others (Moran et al., 2011).

Based on our preliminary findings and those of others, we feel it is imperative to individualize intervention for persons with ASD. Here are some examples: (1) promoting self-advocacy skills based on the decision maker’s dominant thinking style, which is more likely to be deliberative than intuitive for persons with ASD; (2) reframing key life decisions by considering both the possible gains and possible losses; (3) providing encouragement and reassurance as individuals take decision-making risks that are to their advantage; and, (4) providing information for making realistic assessments of others’ decision making as a model for their own processes (e.g., it is sometimes okay to be late for an appointment). By bringing together strengths and complementing these strengths with tools to overcome weaknesses, we believe there is the potential to improve the decisions of individuals on the spectrum and, ultimately, the outcomes of those decisions. For example, our results suggest that high-functioning persons with ASD are quite able to use quantitative and multi-faceted information as decision aids.

### Limitations and Suggestions for Future Research

The conclusions from this study were limited by the following: low sample size (N) for the ASD group, reliance on self-reports for diagnosis, and exploratory choices for tasks, measures, and statistics. We discuss each of these in turn.

The low N for the ASD group was the result of recruiting from a restricted population within a single university and requiring an extensive commitment of time and effort among the participants. In spite of a low N, however, a number of significant findings were obtained. (One step taken to reduce the incidence of Type II errors was to not correct for multiple comparisons.) We hope that this initial degree of success serves as a motivator for future multi-university, multi-collaborator research to multiply the sample size. Of course, expanding the age range and educational status of potential participants would also serve to increase N. However, we believe that the focus on high-functioning college students was warranted in light of the importance of the decisions they are making at this stage of their lives and the impact of these decisions not only for themselves but for society that could benefit from their contributions.

We employed a set of checks and balances for the recruitment of the ASD group. We have confidence that the steps we took were the best we could do under the circumstances and led to few if any false positives. Nevertheless, diagnostic confirmation ultimately depended on participants’ self-reports. To reiterate a point made earlier, if some members of our ASD group were not actually on the spectrum, and, more likely, some in the control groups were on the spectrum, then this would have attenuated our observed group differences. Future research, if it is spread out across institutes, can focus on recruitment from units within the institute that deal specifically with ASD and utilize multiple diagnostic tools.

The tasks and measures we used, because they were exploratory, were somewhat arbitrary. What we learned from this pilot study was which ones hold the most promise for further investigation. This can lead to a narrowing of survey items and the resulting shortening of experimental sessions could aid in recruiting. On the other hand, some of our results suggest measure expansion. For example, risk-taking differences were found between groups but these were confined to a small set of decision domains, specifically monetary gambling type choices for the Cups Task. New research using the multi-domain DOSPERT scale of risk attitude and risk taking (Weber et al., 2002; Blais and Weber, 2006) might be especially interesting because it separates social risks from other types of risks and these may be especially salient for persons with ASD.

Further suggestions for future research include longitudinal or prospective research, especially useful for examining the malleability of decision making skills within the ASD group. Further successful demonstrations should motivate the judgment and decision making research community to apply their tasks and measures to a wider variety of special groups such as persons with Attention Deficit Hyperactivity Disorder, depression, Attachment Disorder, Parkinson’s Disease or Alzheimer’s Disease, each with its own possibly distinct decision making profile. The new knowledge acquired about these profiles would enhance our understanding of special groups and increase the efficacy of interventions and treatments.

## Author Contributions

IL and GG designed the study, oversaw its execution, planned the data analyses, and wrote major sections of the paper. MF was responsible for those portions of the paper dealing with the clinical applications and the associated literature review. CC also contributed to the literature review and aided in participant recruitment and testing. VY helped refine the survey instruments and played a major role in recruitment and testing. HY contributed to data analysis and interpretation, and added valuable insights throughout the project.

### Conflict of Interest Statement

The authors declare that the research was conducted in the absence of any commercial or financial relationships that could be construed as a potential conflict of interest.
