# Corrigendum: Extending decision making competence to special populations: a pilot study of persons on the autism spectrum

**DOI:** 10.3389/fpsyg.2016.00971

**Published:** 2016-07-12

**Authors:** Irwin P. Levin, Gary J. Gaeth, Megan Foley-Nicpon, Vitaliya Yegorova, Charles Cederberg, Haoyang Yan

**Affiliations:** ^1^Department of Psychology, University of IowaIowa City, IA, USA; ^2^Department of Marketing, University of IowaIowa City, IA, USA; ^3^Department of Psychological and Quantitative Foundations, University of IowaIowa City, IA, USA; ^4^Department of Social Work, University of IowaIowa City, IA, USA; ^5^Department of Psychology, University of MichiganAnn Arbor, MI, USA

**Keywords:** decision making, persons on the autism spectrum, risk taking, perception of social norms, framing effects

In the original article we discovered that the code for scoring self-endorsement of social norms was inadvertently reversed for the ASD and Control groups. This led to errors in Table 2 and three incorrect statements in the text.

In Table 2, the control value for “Self-endorsement of Social norms (0–1)” and the control and *t*-values for “Difference between self-endorsement and perceived others' endorsement of Social norms (0–1)” were incorrect. A corrected Table 2 is below:

In the “Results” section, sub-section “Between-group Comparisons,” the last paragraph should now read:

Table 2 focuses on behavioral and social measures: components of Recognizing Social Norms where for each of a series of socially-undesirable acts, participants rate their personal likelihood of saying it is okay to perform each act and estimate the percentage of their peers who would say it is okay. Overall, participants with ASD, compared to controls, gave comparable personal endorsements of behaviors that violate social norms. They also tended to give lower ratings of others' endorsements of these behaviors but this effect failed to reach statistical significance. At the individual item level, participants in the ASD group gave significantly lower ratings than controls for keeping things that don't belong to you, not holding the door open for someone, not being punctual, and not returning calls. Given that participants in the ASD group were as consistent in self-other judgments as controls, their perceptions of others' undesirable behaviors warrants further study.

**Table 2:** ***T*-test results comparing ASD participants with “controls” on Social Measures**.

	**ASD (*N* = 15)**	**Control**	***t*-value (*p*)**
Self-endorsement of Social norms (0–1)	0.38 (0.26)	0.39 (0.20) (*N* = 174)	0.18 (>0.10)
Perception of others' endorsement of Social norms (0–100)	35.69 (17.12)	42.20 (14.11) (*N* = 174)	1.69 (0.0938)
Difference between self-endorsement and perceived others' endorsement of Social norms (0–1)	0.02 (0.22)	–0.03 (0.17) (*N* = 174)	1.09 (0.28)

In the “Discussion” section, sub-section “Summary of Key Results” RQ1 should now read:

Consistent with theory of mind notions that persons on the autism spectrum have difficulties perceiving social cues, individuals in the ASD group tended to be less likely to perceive others as endorsing undesirable social behaviors. The latter was especially true of those with the poorest social functioning. Interestingly, individuals in the ASD group showed a significantly greater degree of coherence between personal endorsements and perceptions of others.

## Conflict of interest statement

The authors declare that the research was conducted in the absence of any commercial or financial relationships that could be construed as a potential conflict of interest.

